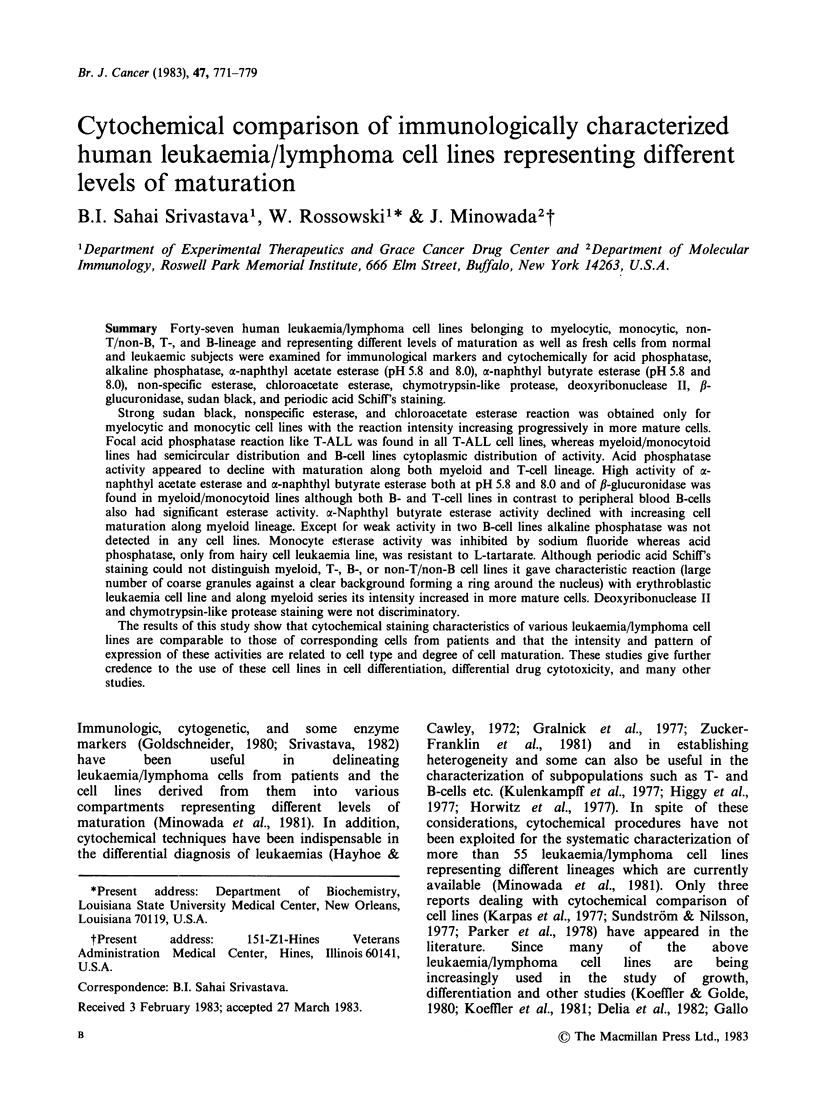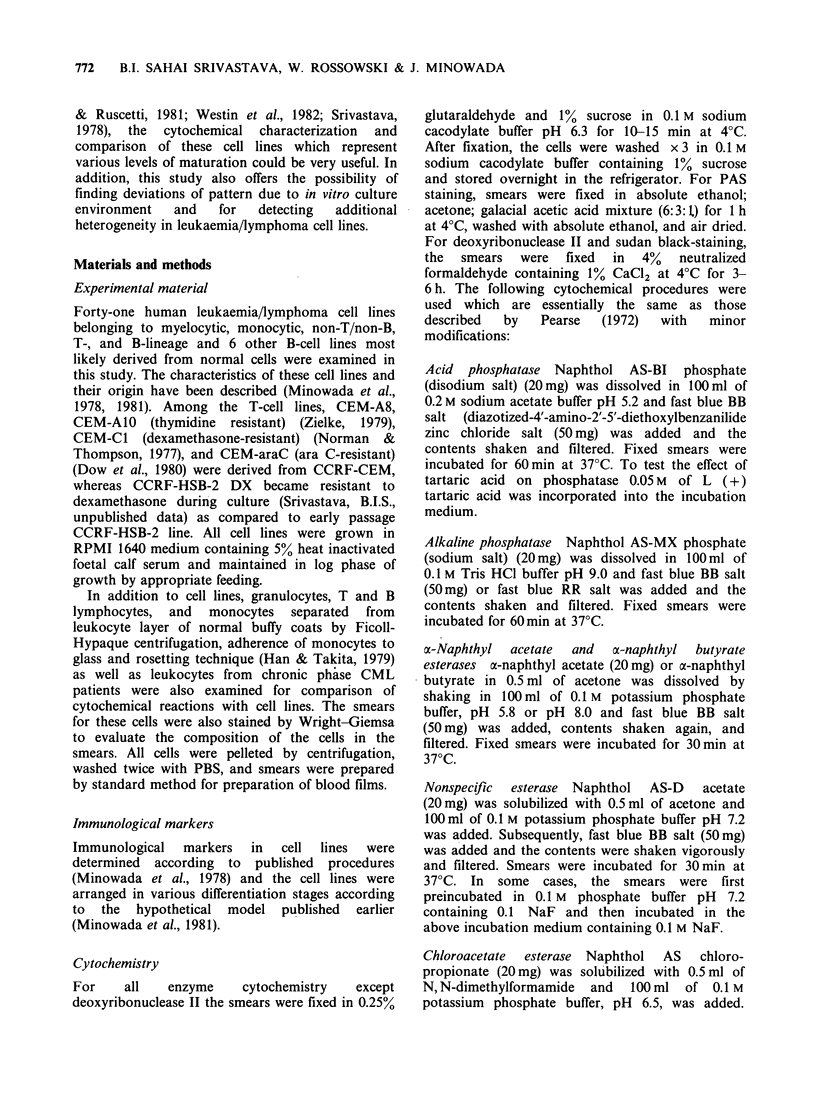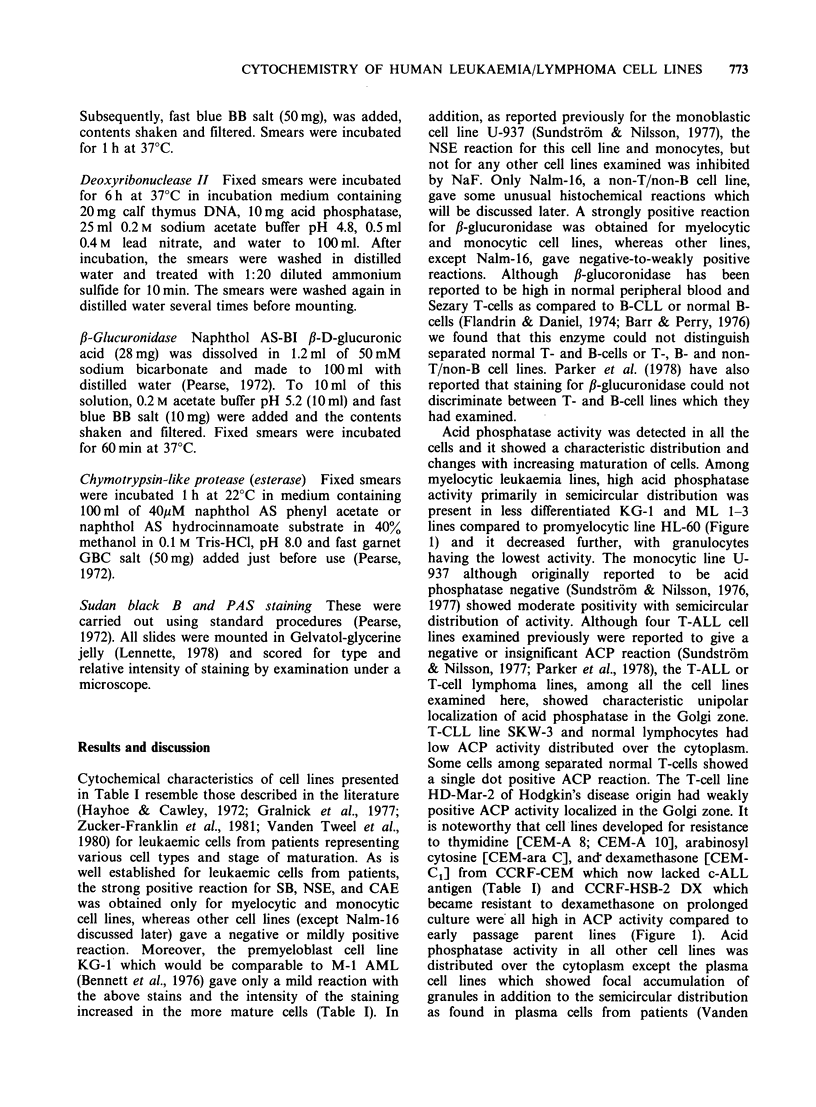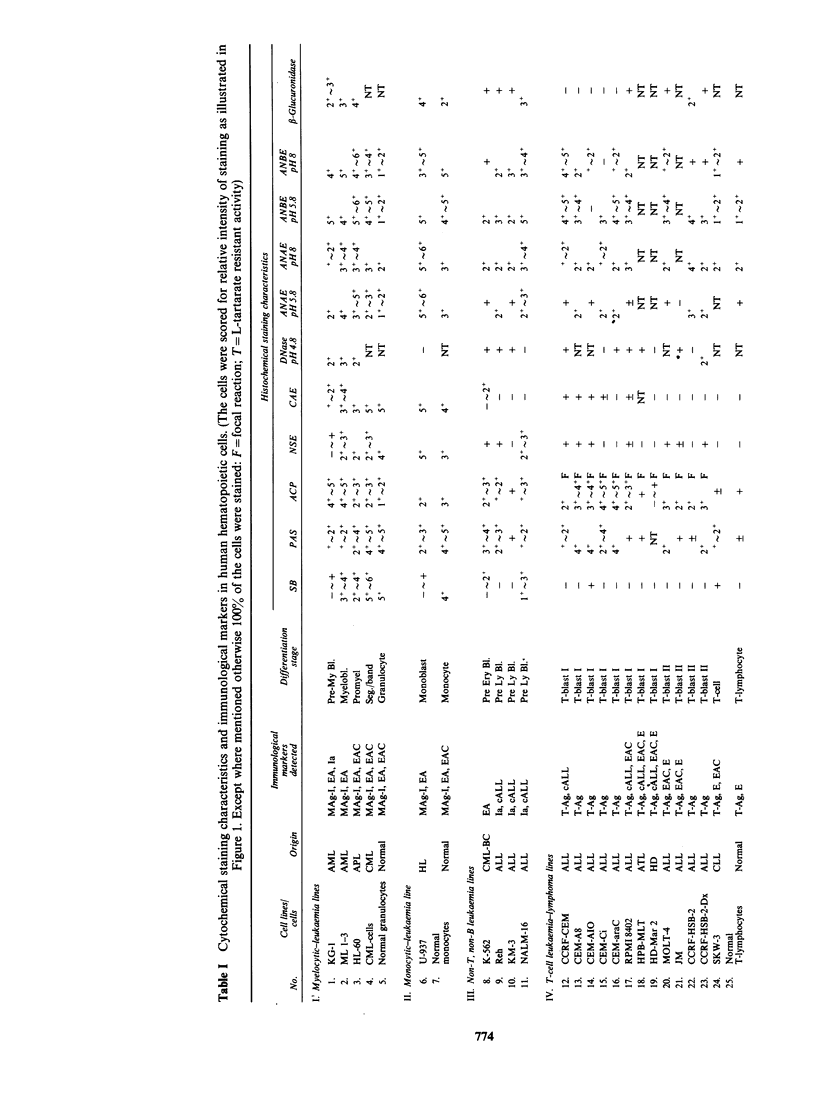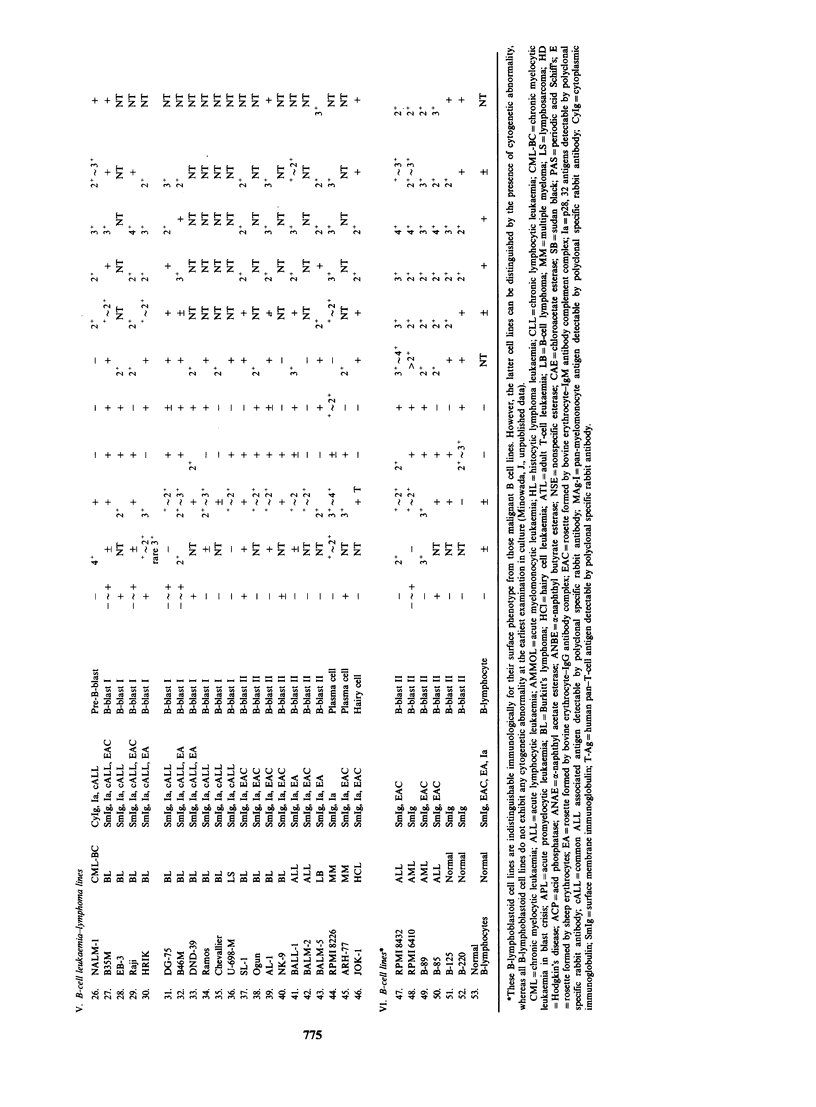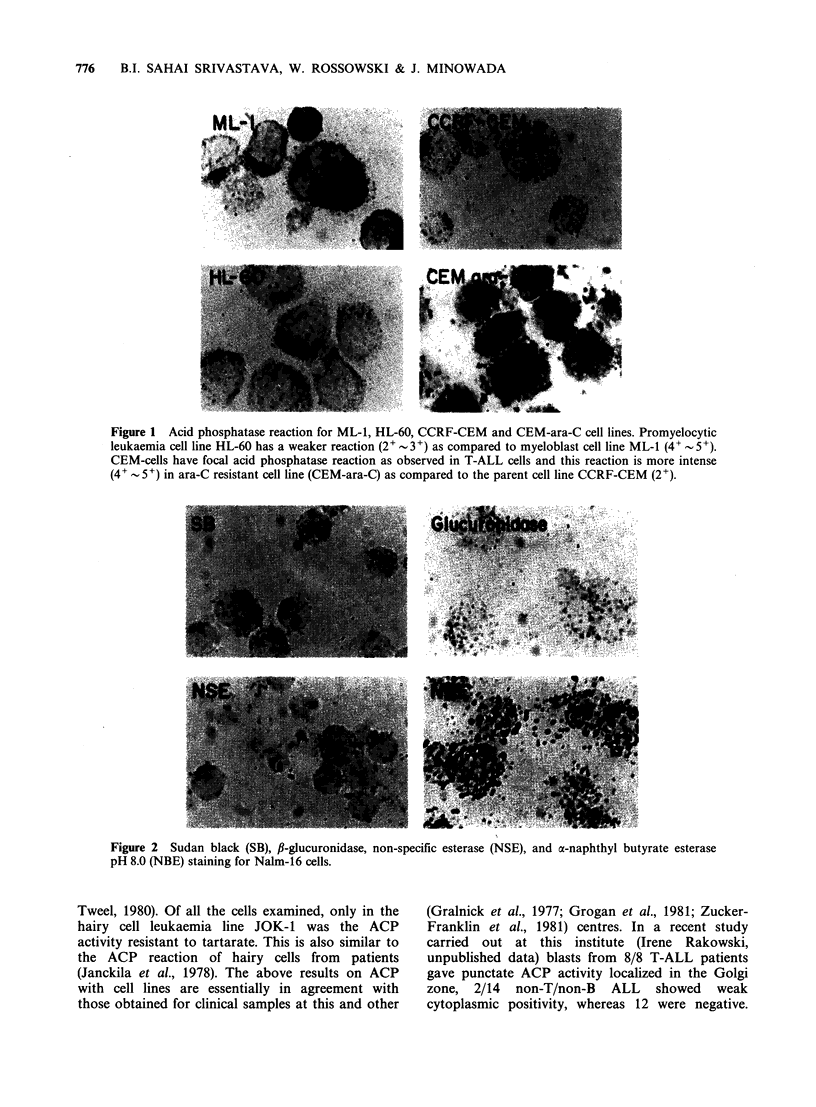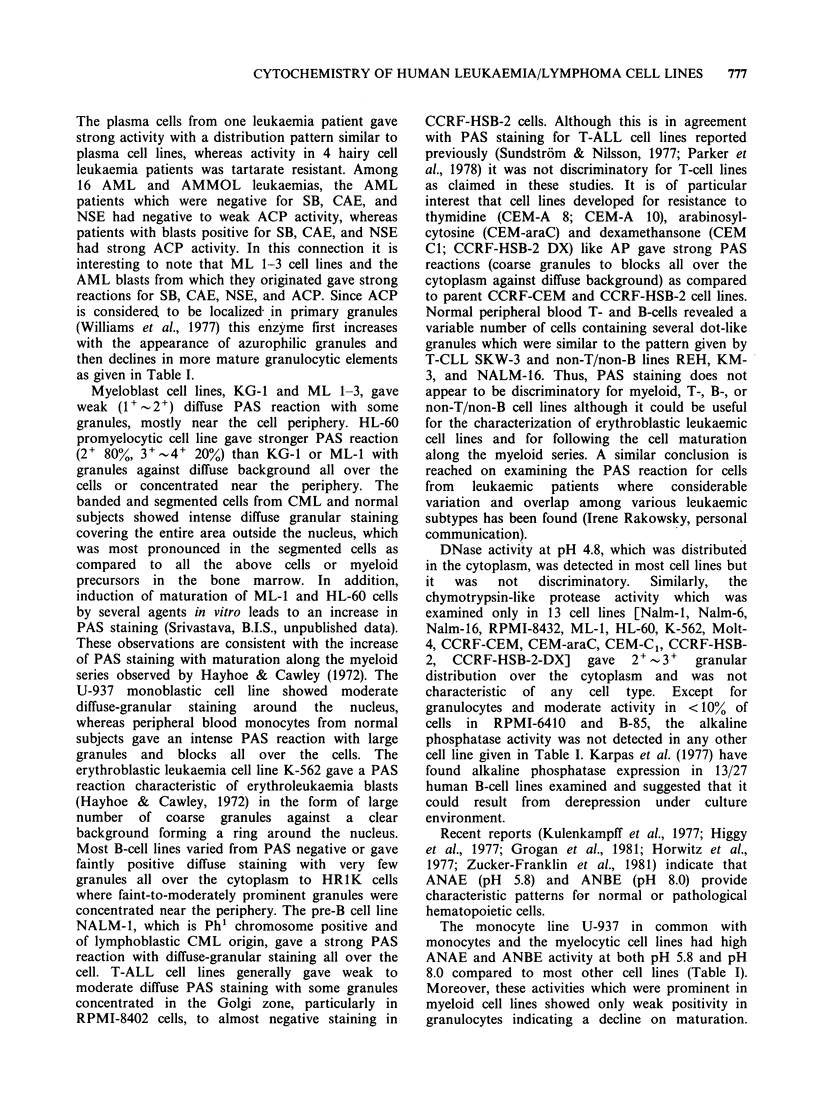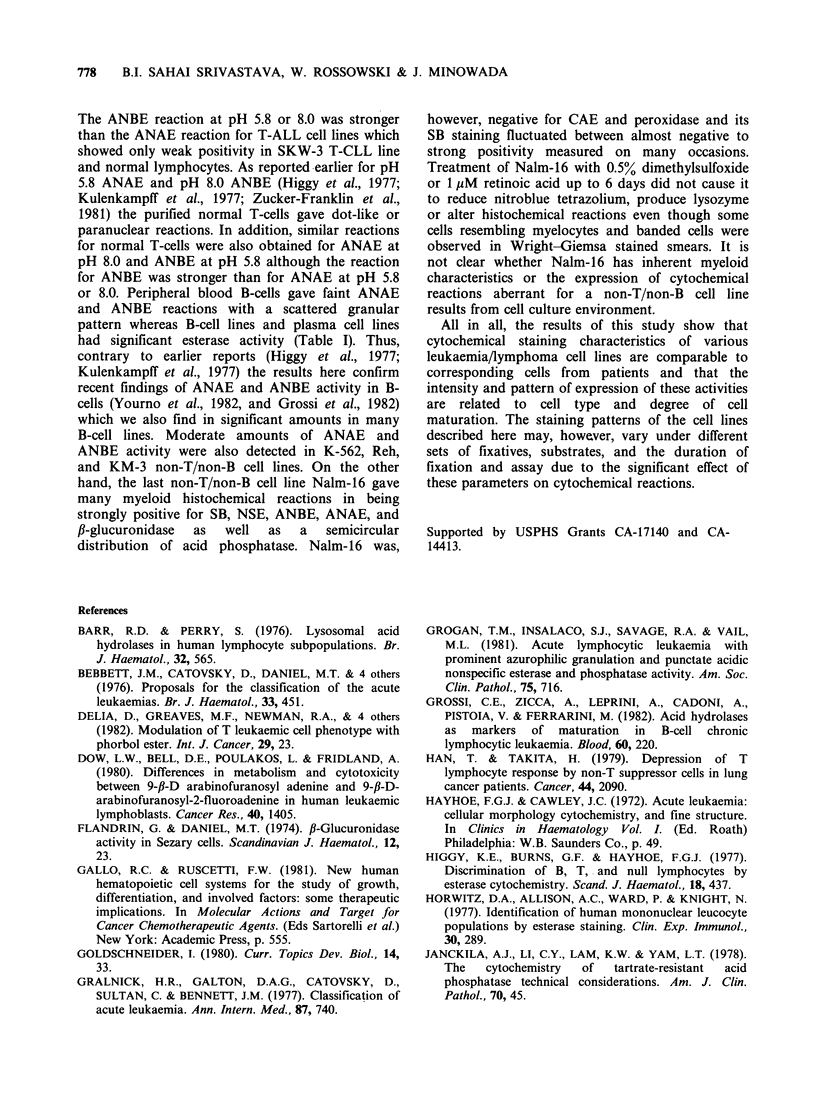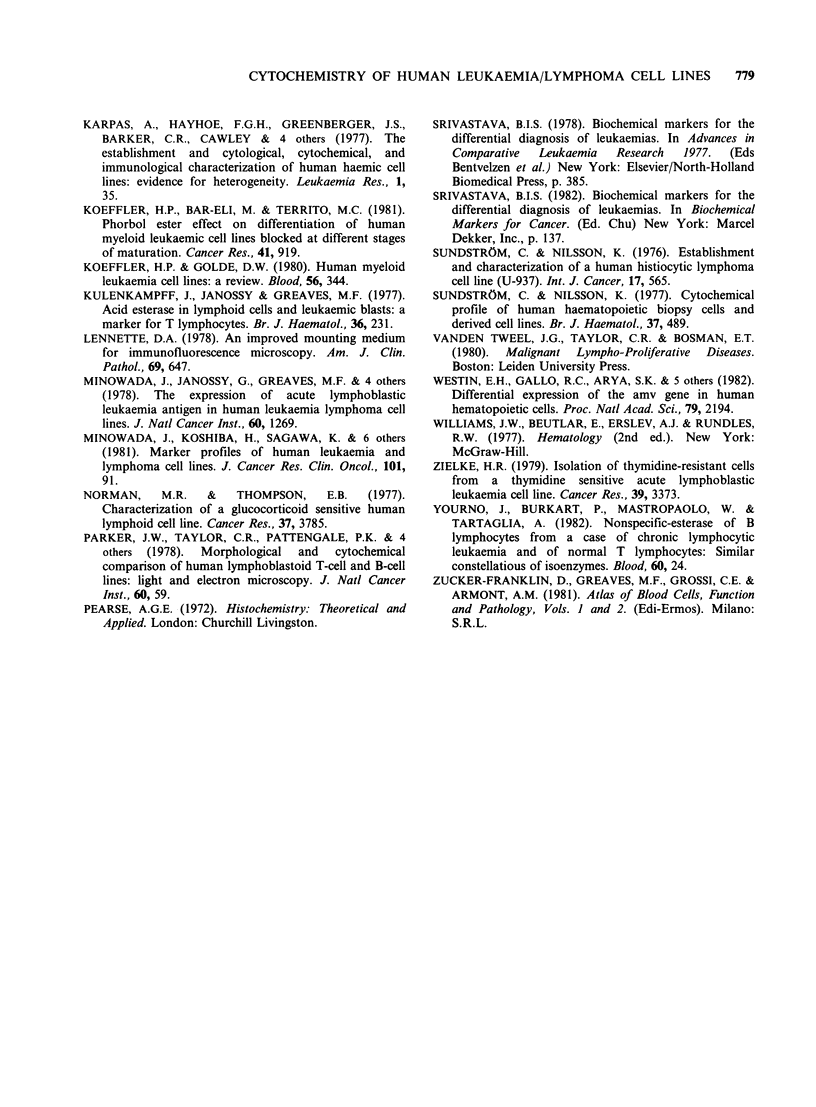# Cytochemical comparison of immunologically characterized human leukaemia/lymphoma cell lines representing different levels of maturation.

**DOI:** 10.1038/bjc.1983.130

**Published:** 1983-06

**Authors:** B. I. Srivastava, W. Rossowski, J. Minowada

## Abstract

**Images:**


Br. J. Cancer (1983), 47, 771–779

### Cytochemical comparison of immunologically characterized human leukaemia/lymphoma cell lines representing different levels of maturation

B.I. Sahai Srivastava[1], W. Rossowski[1]* & J. Minowada[2]†

[1]*Department of Experimental Therapeutics and Grace Cancer Drug Center and* [2]*Department of Molecular Immunology, Roswell Park Memorial Institute, 666 Elm Street, Buffalo, New York 14263, U.S.A.*

**Summary** Forty-seven human leukaemia/lymphoma cell lines belonging to myelocytic, monocytic, non-T/non-B, T-, and B-lineage and representing different levels of maturation as well as fresh cells from normal and leukaemic subjects were examined for immunological markers and cytochemically for acid phosphatase, alkaline phosphatase, α-naphthyl acetate esterase (pH 5.8 and 8.0), α-naphthyl butyrate esterase (pH 5.8 and 8.0), non-specific esterase, chloroacetate esterase, chymotrypsin-like protease, deoxyribonuclease II, β-glucuronidase, sudan black, and periodic acid Schiff's staining.

Strong sudan black, nonspecific esterase, and chloroacetate esterase reaction was obtained only for myelocytic and monocytic cell lines with the reaction intensity increasing progressively in more mature cells. Focal acid phosphatase reaction like T-ALL was found in all T-ALL cell lines, whereas myeloid/monocytoid lines had semicircular distribution and B-cell lines cytoplasmic distribution of activity. Acid phosphatase activity appeared to decline with maturation along both myeloid and T-cell lineage. High activity of α-naphthyl acetate esterase and α-naphthyl butyrate esterase both at pH 5.8 and 8.0 and of β-glucuronidase was found in myeloid/monocytoid lines although both B- and T-cell lines in contrast to peripheral blood B-cells also had significant esterase activity. α-Naphthyl butyrate esterase activity declined with increasing cell maturation along myeloid lineage. Except for weak activity in two B-cell lines alkaline phosphatase was not detected in any cell lines. Monocyte esterase activity was inhibited by sodium fluoride whereas acid phosphatase, only from hairy cell leukaemia line, was resistant to L-tartarate. Although periodic acid Schiff's staining could not distinguish myeloid, T-, B-, or non-T/non-B cell lines it gave characteristic reaction (large number of coarse granules against a clear background forming a ring around the nucleus) with erythroblastic leukaemia cell line and along myeloid series its intensity increased in more mature cells. Deoxyribonuclease II and chymotrypsin-like protease staining were not discriminatory.

The results of this study show that cytochemical staining characteristics of various leukaemia/lymphoma cell lines are comparable to those of corresponding cells from patients and that the intensity and pattern of expression of these activities are related to cell type and degree of cell maturation. These studies give further credence to the use of these cell lines in cell differentiation, differential drug cytotoxicity, and many other studies.

Immunologic, cytogenetic, and some enzyme markers (Goldschneider, 1980; Srivastava, 1982) have been useful in delineating leukaemia/lymphoma cells from patients and the cell lines derived from them into various compartments representing different levels of maturation (Minowada *et al.*, 1981). In addition, cytochemical techniques have been indispensable in the differential diagnosis of leukaemias (Hayhoe & Cawley, 1972; Gralnick *et al.*, 1977; Zucker-Franklin *et al.*, 1981) and in establishing heterogeneity and some can also be useful in the characterization of subpopulations such as T- and B-cells etc. (Kulenkampff *et al.*, 1977; Higgy *et al.*, 1977; Horwitz *et al.*, 1977). In spite of these considerations, cytochemical procedures have not been exploited for the systematic characterization of more than 55 leukaemia/lymphoma cell lines representing different lineages which are currently available (Minowada *et al.*, 1981). Only three reports dealing with cytochemical comparison of cell lines (Karpas *et al.*, 1977; Sundström & Nilsson, 1977; Parker *et al.*, 1978) have appeared in the literature. Since many of the above leukaemia/lymphoma cell lines are being increasingly used in the study of growth, differentiation and other studies (Koeffler & Golde, 1980; Koeffler *et al.*, 1981; Delia *et al.*, 1982; Gallo

*Present address: Department of Biochemistry, Louisiana State University Medical Center, New Orleans, Louisiana 70119, U.S.A.

†Present address: 151-Z1-Hines Veterans Administration Medical Center, Hines, Illinois 60141, U.S.A.

Correspondence: B.I. Sahai Srivastava.

Received 3 February 1983; accepted 27 March 1983.

© The Macmillan Press Ltd., 1983

& Ruscetti, 1981; Westin *et al.*, 1982; Srivastava, 1978), the cytochemical characterization and comparison of these cell lines which represent various levels of maturation could be very useful. In addition, this study also offers the possibility of finding deviations of pattern due to *in vitro* culture environment and for detecting additional heterogeneity in leukaemia/lymphoma cell lines.

### Materials and methods

### *Experimental material*

Forty-one human leukaemia/lymphoma cell lines belonging to myelocytic, monocytic, non-T/non-B, T-, and B-lineage and 6 other B-cell lines most likely derived from normal cells were examined in this study. The characteristics of these cell lines and their origin have been described (Minowada *et al.*, 1978, 1981). Among the T-cell lines, CEM-A8, CEM-A10 (thymidine resistant) (Zielke, 1979), CEM-C1 (dexamethasone-resistant) (Norman & Thompson, 1977), and CEM-araC (ara C-resistant) (Dow *et al.*, 1980) were derived from CCRF-CEM, whereas CCRF-HSB-2 DX became resistant to dexamethasone during culture (Srivastava, B.I.S., unpublished data) as compared to early passage CCRF-HSB-2 line. All cell lines were grown in RPMI 1640 medium containing 5% heat inactivated foetal calf serum and maintained in log phase of growth by appropriate feeding.

In addition to cell lines, granulocytes, T and B lymphocytes, and monocytes separated from leukocyte layer of normal buffy coats by Ficoll-Hypaque centrifugation, adherence of monocytes to glass and rosetting technique (Han & Takita, 1979) as well as leukocytes from chronic phase CML patients were also examined for comparison of cytochemical reactions with cell lines. The smears for these cells were also stained by Wright–Giemsa to evaluate the composition of the cells in the smears. All cells were pelleted by centrifugation, washed twice with PBS, and smears were prepared by standard method for preparation of blood films.

### *Immunological markers*

Immunological markers in cell lines were determined according to published procedures (Minowada *et al.*, 1978) and the cell lines were arranged in various differentiation stages according to the hypothetical model published earlier (Minowada *et al.*, 1981).

### *Cytochemistry*

For all enzyme cytochemistry except deoxyribonuclease II the smears were fixed in 0.25% glutaraldehyde and 1% sucrose in 0.1 M sodium cacodylate buffer pH 6.3 for 10–15 min at 4°C. After fixation, the cells were washed ×3 in 0.1 M sodium cacodylate buffer containing 1% sucrose and stored overnight in the refrigerator. For PAS staining, smears were fixed in absolute ethanol; acetone; galacial acetic acid mixture (6:3:1) for 1 h at 4°C, washed with absolute ethanol, and air dried. For deoxyribonuclease II and sudan black-staining, the smears were fixed in 4% neutralized formaldehyde containing 1% $CaCl_2$ at 4°C for 3–6 h. The following cytochemical procedures were used which are essentially the same as those described by Pearse (1972) with minor modifications:

*Acid phosphatase* Naphthol AS-BI phosphate (disodium salt) (20 mg) was dissolved in 100 ml of 0.2 M sodium acetate buffer pH 5.2 and fast blue BB salt (diazotized-4′-amino-2′-5′-diethoxylbenzanilide zinc chloride salt (50 mg) was added and the contents shaken and filtered. Fixed smears were incubated for 60 min at 37°C. To test the effect of tartaric acid on phosphatase 0.05 M of L (+) tartaric acid was incorporated into the incubation medium.

*Alkaline phosphatase* Naphthol AS-MX phosphate (sodium salt) (20 mg) was dissolved in 100 ml of 0.1 M Tris HCl buffer pH 9.0 and fast blue BB salt (50 mg) or fast blue RR salt was added and the contents shaken and filtered. Fixed smears were incubated for 60 min at 37°C.

*α-Naphthyl acetate and α-naphthyl butyrate esterases* α-naphthyl acetate (20 mg) or α-naphthyl butyrate in 0.5 ml of acetone was dissolved by shaking in 100 ml of 0.1 M potassium phosphate buffer, pH 5.8 or pH 8.0 and fast blue BB salt (50 mg) was added, contents shaken again, and filtered. Fixed smears were incubated for 30 min at 37°C.

*Nonspecific esterase* Naphthol AS-D acetate (20 mg) was solubilized with 0.5 ml of acetone and 100 ml of 0.1 M potassium phosphate buffer pH 7.2 was added. Subsequently, fast blue BB salt (50 mg) was added and the contents were shaken vigorously and filtered. Smears were incubated for 30 min at 37°C. In some cases, the smears were first preincubated in 0.1 M phosphate buffer pH 7.2 containing 0.1 NaF and then incubated in the above incubation medium containing 0.1 M NaF.

*Chloroacetate esterase* Naphthol AS chloropropionate (20 mg) was solubilized with 0.5 ml of N,N-dimethylformamide and 100 ml of 0.1 M potassium phosphate buffer, pH 6.5, was added.

Subsequently, fast blue BB salt (50 mg), was added, contents shaken and filtered. Smears were incubated for 1 h at 37°C.

*Deoxyribonuclease II* Fixed smears were incubated for 6 h at 37°C in incubation medium containing 20 mg calf thymus DNA, 10 mg acid phosphatase, 25 ml 0.2 M sodium acetate buffer pH 4.8, 0.5 ml 0.4 M lead nitrate, and water to 100 ml. After incubation, the smears were washed in distilled water and treated with 1:20 diluted ammonium sulfide for 10 min. The smears were washed again in distilled water several times before mounting.

*β-Glucuronidase* Naphthol AS-BI β-D-glucuronic acid (28 mg) was dissolved in 1.2 ml of 50 mM sodium bicarbonate and made to 100 ml with distilled water (Pearse, 1972). To 10 ml of this solution, 0.2 M acetate buffer pH 5.2 (10 ml) and fast blue BB salt (10 mg) were added and the contents shaken and filtered. Fixed smears were incubated for 60 min at 37°C.

*Chymotrypsin-like protease (esterase)* Fixed smears were incubated 1 h at 22°C in medium containing 100 ml of 40μM naphthol AS phenyl acetate or naphthol AS hydrocinnamoate substrate in 40% methanol in 0.1 M Tris-HCl, pH 8.0 and fast garnet GBC salt (50 mg) added just before use (Pearse, 1972).

*Sudan black B and PAS staining* These were carried out using standard procedures (Pearse, 1972). All slides were mounted in Gelvatol-glycerine jelly (Lennette, 1978) and scored for type and relative intensity of staining by examination under a microscope.

### Results and discussion

Cytochemical characteristics of cell lines presented in Table I resemble those described in the literature (Hayhoe & Cawley, 1972; Gralnick *et al.*, 1977; Zucker-Franklin *et al.*, 1981; Vanden Tweel *et al.*, 1980) for leukaemic cells from patients representing various cell types and stage of maturation. As is well established for leukaemic cells from patients, the strong positive reaction for SB, NSE, and CAE was obtained only for myelocytic and monocytic cell lines, whereas other cell lines (except Nalm-16 discussed later) gave a negative or mildly positive reaction. Moreover, the premyeloblast cell line KG-1 which would be comparable to M-1 AML (Bennett *et al.*, 1976) gave only a mild reaction with the above stains and the intensity of the staining increased in the more mature cells (Table I). In addition, as reported previously for the monoblastic cell line U-937 (Sundström & Nilsson, 1977), the NSE reaction for this cell line and monocytes, but not for any other cell lines examined was inhibited by NaF. Only Nalm-16, a non-T/non-B cell line, gave some unusual histochemical reactions which will be discussed later. A strongly positive reaction for β-glucuronidase was obtained for myelocytic and monocytic cell lines, whereas other lines, except Nalm-16, gave negative-to-weakly positive reactions. Although β-glucoronidase has been reported to be high in normal peripheral blood and Sezary T-cells as compared to B-CLL or normal B-cells (Flandrin & Daniel, 1974; Barr & Perry, 1976) we found that this enzyme could not distinguish separated normal T- and B-cells or T-, B- and non-T/non-B cell lines. Parker *et al.* (1978) have also reported that staining for β-glucuronidase could not discriminate between T- and B-cell lines which they had examined.

Acid phosphatase activity was detected in all the cells and it showed a characteristic distribution and changes with increasing maturation of cells. Among myelocytic leukaemia lines, high acid phosphatase activity primarily in semicircular distribution was present in less differentiated KG-1 and ML 1–3 lines compared to promyelocytic line HL-60 (Figure 1) and it decreased further, with granulocytes having the lowest activity. The monocytic line U-937 although originally reported to be acid phosphatase negative (Sundström & Nilsson, 1976, 1977) showed moderate positivity with semicircular distribution of activity. Although four T-ALL cell lines examined previously were reported to give a negative or insignificant ACP reaction (Sundström & Nilsson, 1977; Parker *et al.*, 1978), the T-ALL or T-cell lymphoma lines, among all the cell lines examined here, showed characteristic unipolar localization of acid phosphatase in the Golgi zone. T-CLL line SKW-3 and normal lymphocytes had low ACP activity distributed over the cytoplasm. Some cells among separated normal T-cells showed a single dot positive ACP reaction. The T-cell line HD-Mar-2 of Hodgkin's disease origin had weakly positive ACP activity localized in the Golgi zone. It is noteworthy that cell lines developed for resistance to thymidine [CEM-A 8; CEM-A 10], arabinosyl cytosine [CEM-ara C], and dexamethasone [CEM-$C_1$] from CCRF-CEM which now lacked c-ALL antigen (Table I) and CCRF-HSB-2 DX which became resistant to dexamethasone on prolonged culture were all high in ACP activity compared to early passage parent lines (Figure 1). Acid phosphatase activity in all other cell lines was distributed over the cytoplasm except the plasma cell lines which showed focal accumulation of granules in addition to the semicircular distribution as found in plasma cells from patients (Vanden

**Figure 1** Acid phosphatase reaction for ML-1, HL-60, CCRF-CEM and CEM-ara-C cell lines. Promyelocytic leukaemia cell line HL-60 has a weaker reaction ($2^+ \sim 3^+$) as compared to myeloblast cell line ML-1 ($4^+ \sim 5^+$). CEM-cells have focal acid phosphatase reaction as observed in T-ALL cells and this reaction is more intense ($4^+ \sim 5^+$) in ara-C resistant cell line (CEM-ara-C) as compared to the parent cell line CCRF-CEM ($2^+$).

**Figure 2** Sudan black (SB), $\beta$-glucuronidase, non-specific esterase (NSE), and $\alpha$-naphthyl butyrate esterase pH 8.0 (NBE) staining for Nalm-16 cells.

Tweel, 1980). Of all the cells examined, only in the hairy cell leukaemia line JOK-1 was the ACP activity resistant to tartarate. This is also similar to the ACP reaction of hairy cells from patients (Janckila *et al.*, 1978). The above results on ACP with cell lines are essentially in agreement with those obtained for clinical samples at this and other (Gralnick *et al.*, 1977; Grogan *et al.*, 1981; Zucker-Franklin *et al.*, 1981) centres. In a recent study carried out at this institute (Irene Rakowski, unpublished data) blasts from 8/8 T-ALL patients gave punctate ACP activity localized in the Golgi zone, 2/14 non-T/non-B ALL showed weak cytoplasmic positivity, whereas 12 were negative.

The plasma cells from one leukaemia patient gave strong activity with a distribution pattern similar to plasma cell lines, whereas activity in 4 hairy cell leukaemia patients was tartarate resistant. Among 16 AML and AMMOL leukaemias, the AML patients which were negative for SB, CAE, and NSE had negative to weak ACP activity, whereas patients with blasts positive for SB, CAE, and NSE had strong ACP activity. In this connection it is interesting to note that ML 1–3 cell lines and the AML blasts from which they originated gave strong reactions for SB, CAE, NSE, and ACP. Since ACP is considered to be localized in primary granules (Williams *et al.*, 1977) this enzyme first increases with the appearance of azurophilic granules and then declines in more mature granulocytic elements as given in Table I.

Myeloblast cell lines, KG-1 and ML 1–3, gave weak ($1^+ \sim 2^+$) diffuse PAS reaction with some granules, mostly near the cell periphery. HL-60 promyelocytic cell line gave stronger PAS reaction ($2^+$ 80%, $3^+ \sim 4^+$ 20%) than KG-1 or ML-1 with granules against diffuse background all over the cells or concentrated near the periphery. The banded and segmented cells from CML and normal subjects showed intense diffuse granular staining covering the entire area outside the nucleus, which was most pronounced in the segmented cells as compared to all the above cells or myeloid precursors in the bone marrow. In addition, induction of maturation of ML-1 and HL-60 cells by several agents *in vitro* leads to an increase in PAS staining (Srivastava, B.I.S., unpublished data). These observations are consistent with the increase of PAS staining with maturation along the myeloid series observed by Hayhoe & Cawley (1972). The U-937 monoblastic cell line showed moderate diffuse-granular staining around the nucleus, whereas peripheral blood monocytes from normal subjects gave an intense PAS reaction with large granules and blocks all over the cells. The erythroblastic leukaemia cell line K-562 gave a PAS reaction characteristic of erythroleukaemia blasts (Hayhoe & Cawley, 1972) in the form of large number of coarse granules against a clear background forming a ring around the nucleus. Most B-cell lines varied from PAS negative or gave faintly positive diffuse staining with very few granules all over the cytoplasm to HR1K cells where faint-to-moderately prominent granules were concentrated near the periphery. The pre-B cell line NALM-1, which is $Ph^1$ chromosome positive and of lymphoblastic CML origin, gave a strong PAS reaction with diffuse-granular staining all over the cell. T-ALL cell lines generally gave weak to moderate diffuse PAS staining with some granules concentrated in the Golgi zone, particularly in RPMI-8402 cells, to almost negative staining in CCRF-HSB-2 cells. Although this is in agreement with PAS staining for T-ALL cell lines reported previously (Sundström & Nilsson, 1977; Parker *et al.*, 1978) it was not discriminatory for T-cell lines as claimed in these studies. It is of particular interest that cell lines developed for resistance to thymidine (CEM-A 8; CEM-A 10), arabinosyl-cytosine (CEM-araC) and dexamethansone (CEM C1; CCRF-HSB-2 DX) like AP gave strong PAS reactions (coarse granules to blocks all over the cytoplasm against diffuse background) as compared to parent CCRF-CEM and CCRF-HSB-2 cell lines. Normal peripheral blood T- and B-cells revealed a variable number of cells containing several dot-like granules which were similar to the pattern given by T-CLL SKW-3 and non-T/non-B lines REH, KM-3, and NALM-16. Thus, PAS staining does not appear to be discriminatory for myeloid, T-, B-, or non-T/non-B cell lines although it could be useful for the characterization of erythroblastic leukaemic cell lines and for following the cell maturation along the myeloid series. A similar conclusion is reached on examining the PAS reaction for cells from leukaemic patients where considerable variation and overlap among various leukaemic subtypes has been found (Irene Rakowsky, personal communication).

DNase activity at pH 4.8, which was distributed in the cytoplasm, was detected in most cell lines but it was not discriminatory. Similarly, the chymotrypsin-like protease activity which was examined only in 13 cell lines [Nalm-1, Nalm-6, Nalm-16, RPMI-8432, ML-1, HL-60, K-562, Molt-4, CCRF-CEM, CEM-araC, CEM-$C_1$, CCRF-HSB-2, CCRF-HSB-2-DX] gave $2^+ \sim 3^+$ granular distribution over the cytoplasm and was not characteristic of any cell type. Except for granulocytes and moderate activity in $<10\%$ of cells in RPMI-6410 and B-85, the alkaline phosphatase activity was not detected in any other cell line given in Table I. Karpas *et al.* (1977) have found alkaline phosphatase expression in 13/27 human B-cell lines examined and suggested that it could result from derepression under culture environment.

Recent reports (Kulenkampff *et al.*, 1977; Higgy *et al.*, 1977; Grogan *et al.*, 1981; Horwitz *et al.*, 1977; Zucker-Franklin *et al.*, 1981) indicate that ANAE (pH 5.8) and ANBE (pH 8.0) provide characteristic patterns for normal or pathological hematopoietic cells.

The monocyte line U-937 in common with monocytes and the myelocytic cell lines had high ANAE and ANBE activity at both pH 5.8 and pH 8.0 compared to most other cell lines (Table I). Moreover, these activities which were prominent in myeloid cell lines showed only weak positivity in granulocytes indicating a decline on maturation.

The ANBE reaction at pH 5.8 or 8.0 was stronger than the ANAE reaction for T-ALL cell lines which showed only weak positivity in SKW-3 T-CLL line and normal lymphocytes. As reported earlier for pH 5.8 ANAE and pH 8.0 ANBE (Higgy *et al.*, 1977; Kulenkampff *et al.*, 1977; Zucker-Franklin *et al.*, 1981) the purified normal T-cells gave dot-like or paranuclear reactions. In addition, similar reactions for normal T-cells were also obtained for ANAE at pH 8.0 and ANBE at pH 5.8 although the reaction for ANBE was stronger than for ANAE at pH 5.8 or 8.0. Peripheral blood B-cells gave faint ANAE and ANBE reactions with a scattered granular pattern whereas B-cell lines and plasma cell lines had significant esterase activity (Table I). Thus, contrary to earlier reports (Higgy *et al.*, 1977; Kulenkampff *et al.*, 1977) the results here confirm recent findings of ANAE and ANBE activity in B-cells (Yourno *et al.*, 1982, and Grossi *et al.*, 1982) which we also find in significant amounts in many B-cell lines. Moderate amounts of ANAE and ANBE activity were also detected in K-562, Reh, and KM-3 non-T/non-B cell lines. On the other hand, the last non-T/non-B cell line Nalm-16 gave many myeloid histochemical reactions in being strongly positive for SB, NSE, ANBE, ANAE, and $\beta$-glucuronidase as well as a semicircular distribution of acid phosphatase. Nalm-16 was, however, negative for CAE and peroxidase and its SB staining fluctuated between almost negative to strong positivity measured on many occasions. Treatment of Nalm-16 with 0.5% dimethylsulfoxide or 1 $\mu$M retinoic acid up to 6 days did not cause it to reduce nitroblue tetrazolium, produce lysozyme or alter histochemical reactions even though some cells resembling myelocytes and banded cells were observed in Wright–Giemsa stained smears. It is not clear whether Nalm-16 has inherent myeloid characteristics or the expression of cytochemical reactions aberrant for a non-T/non-B cell line results from cell culture environment.

All in all, the results of this study show that cytochemical staining characteristics of various leukaemia/lymphoma cell lines are comparable to corresponding cells from patients and that the intensity and pattern of expression of these activities are related to cell type and degree of cell maturation. The staining patterns of the cell lines described here may, however, vary under different sets of fixatives, substrates, and the duration of fixation and assay due to the significant effect of these parameters on cytochemical reactions.

Supported by USPHS Grants CA-17140 and CA-14413.

**References**

BARR, R.D. & PERRY, S. (1976). Lysosomal acid hydrolases in human lymphocyte subpopulations. *Br. J. Haematol.*, **32,** 565.

BEBBETT, J.M., CATOVSKY, D., DANIEL, M.T. & 4 others (1976). Proposals for the classification of the acute leukaemias. *Br. J. Haematol.*, **33,** 451.

DELIA, D., GREAVES, M.F., NEWMAN, R.A., & 4 others (1982). Modulation of T leukaemic cell phenotype with phorbol ester. *Int. J. Cancer*, **29,** 23.

DOW, L.W., BELL, D.E., POULAKOS, L. & FRIDLAND, A. (1980). Differences in metabolism and cytotoxicity between 9-$\beta$-D arabinofuranosyl adenine and 9-$\beta$-D-arabinofuranosyl-2-fluoroadenine in human leukaemic lymphoblasts. *Cancer Res.*, **40,** 1405.

FLANDRIN, G. & DANIEL, M.T. (1974). $\beta$-Glucuronidase activity in Sezary cells. *Scandinavian J. Haematol.*, **12,** 23.

GALLO, R.C. & RUSCETTI, F.W. (1981). New human hematopoietic cell systems for the study of growth, differentiation, and involved factors: some therapeutic implications. In *Molecular Actions and Target for Cancer Chemotherapeutic Agents.* (Eds Sartorelli *et al.*) New York: Academic Press, p. 555.

GOLDSCHNEIDER, I. (1980). *Curr. Topics Dev. Biol.*, **14,** 33.

GRALNICK, H.R., GALTON, D.A.G., CATOVSKY, D., SULTAN, C. & BENNETT, J.M. (1977). Classification of acute leukaemia. *Ann. Intern. Med.*, **87,** 740.

GROGAN, T.M., INSALACO, S.J., SAVAGE, R.A. & VAIL, M.L. (1981). Acute lymphocytic leukaemia with prominent azurophilic granulation and punctate acidic nonspecific esterase and phosphatase activity. *Am. Soc. Clin. Pathol.*, **75,** 716.

GROSSI, C.E., ZICCA, A., LEPRINI, A., CADONI, A., PISTOIA, V. & FERRARINI, M. (1982). Acid hydrolases as markers of maturation in B-cell chronic lymphocytic leukaemia. *Blood*, **60,** 220.

HAN, T. & TAKITA, H. (1979). Depression of T lymphocyte response by non-T suppressor cells in lung cancer patients. *Cancer*, **44,** 2090.

HAYHOE, F.G.J. & CAWLEY, J.C. (1972). Acute leukaemia: cellular morphology cytochemistry, and fine structure. In *Clinics in Haematology Vol. I.* (Ed. Roath) Philadelphia: W.B. Saunders Co., p. 49.

HIGGY, K.E., BURNS, G.F. & HAYHOE, F.G.J. (1977). Discrimination of B, T, and null lymphocytes by esterase cytochemistry. *Scand. J. Haematol.*, **18,** 437.

HORWITZ, D.A., ALLISON, A.C., WARD, P. & KNIGHT, N. (1977). Identification of human mononuclear leucocyte populations by esterase staining. *Clin. Exp. Immunol.*, **30,** 289.

JANCKILA, A.J., LI, C.Y., LAM, K.W. & YAM, L.T. (1978). The cytochemistry of tartrate-resistant acid phosphatase technical considerations. *Am. J. Clin. Pathol.*, **70,** 45.

KARPAS, A., HAYHOE, F.G.H., GREENBERGER, J.S., BARKER, C.R., CAWLEY & 4 others (1977). The establishment and cytological, cytochemical, and immunological characterization of human haemic cell lines: evidence for heterogeneity. *Leukaemia Res.*, **1**, 35.

KOEFFLER, H.P., BAR-ELI, M. & TERRITO, M.C. (1981). Phorbol ester effect on differentiation of human myeloid leukaemic cell lines blocked at different stages of maturation. *Cancer Res.*, **41**, 919.

KOEFFLER, H.P. & GOLDE, D.W. (1980). Human myeloid leukaemia cell lines: a review. *Blood*, **56**, 344.

KULENKAMPFF, J., JANOSSY & GREAVES, M.F. (1977). Acid esterase in lymphoid cells and leukaemic blasts: a marker for T lymphocytes. *Br. J. Haematol.*, **36**, 231.

LENNETTE, D.A. (1978). An improved mounting medium for immunofluorescence microscopy. *Am. J. Clin. Pathol.*, **69**, 647.

MINOWADA, J., JANOSSY, G., GREAVES, M.F. & 4 others (1978). The expression of acute lymphoblastic leukaemia antigen in human leukaemia lymphoma cell lines. *J. Natl Cancer Inst.*, **60**, 1269.

MINOWADA, J., KOSHIBA, H., SAGAWA, K. & 6 others (1981). Marker profiles of human leukaemia and lymphoma cell lines. *J. Cancer Res. Clin. Oncol.*, **101**, 91.

NORMAN, M.R. & THOMPSON, E.B. (1977). Characterization of a glucocorticoid sensitive human lymphoid cell line. *Cancer Res.*, **37**, 3785.

PARKER, J.W., TAYLOR, C.R., PATTENGALE, P.K. & 4 others (1978). Morphological and cytochemical comparison of human lymphoblastoid T-cell and B-cell lines: light and electron microscopy. *J. Natl Cancer Inst.*, **60**, 59.

PEARSE, A.G.E. (1972). *Histochemistry: Theoretical and Applied*. London: Churchill Livingston.

SRIVASTAVA, B.I.S. (1978). Biochemical markers for the differential diagnosis of leukaemias. In *Advances in Comparative Leukaemia Research 1977*. (Eds Bentvelzen *et al.)* New York: Elsevier/North-Holland Biomedical Press, p. 385.

SRIVASTAVA, B.I.S. (1982). Biochemical markers for the differential diagnosis of leukaemias. In *Biochemical Markers for Cancer*. (Ed. Chu) New York: Marcel Dekker, Inc., p. 137.

SUNDSTRÖM, C. & NILSSON, K. (1976). Establishment and characterization of a human histiocytic lymphoma cell line (U-937). *Int. J. Cancer*, **17**, 565.

SUNDSTRÖM, C. & NILSSON, K. (1977). Cytochemical profile of human haematopoietic biopsy cells and derived cell lines. *Br. J. Haematol.*, **37**, 489.

VANDEN TWEEL, J.G., TAYLOR, C.R. & BOSMAN, E.T. (1980). *Malignant Lympho-Proliferative Diseases*. Boston: Leiden University Press.

WESTIN, E.H., GALLO, R.C., ARYA, S.K. & 5 others (1982). Differential expression of the amv gene in human hematopoietic cells. *Proc. Natl Acad. Sci.*, **79**, 2194.

WILLIAMS, J.W., BEUTLAR, E., ERSLEV, A.J. & RUNDLES, R.W. (1977). *Hematology* (2nd ed.). New York: McGraw-Hill.

ZIELKE, H.R. (1979). Isolation of thymidine-resistant cells from a thymidine sensitive acute lymphoblastic leukaemia cell line. *Cancer Res.*, **39**, 3373.

YOURNO, J., BURKART, P., MASTROPAOLO, W. & TARTAGLIA, A. (1982). Nonspecific-esterase of B lymphocytes from a case of chronic lymphocytic leukaemia and of normal T lymphocytes: Similar constellatious of isoenzymes. *Blood*, **60**, 24.

ZUCKER-FRANKLIN, D., GREAVES, M.F., GROSSI, C.E. & ARMONT, A.M. (1981). *Atlas of Blood Cells, Function and Pathology, Vols. 1 and 2*. (Edi-Ermos). Milano: S.R.L.